# Supplementary material for: Splice-Junction-Based Mapping of Alternative Isoforms in the Human Proteome
Source: Cell Rep. Author manuscript; Available in PMC 2020 Jan 15. (PMC6961840; doi:10.1016/j.celrep.2019.11.026)
Supplement: 2 [file NIHMS1546469-supplement-2.zip › DF1/PXD010154/Thyroid/Thyroid_25_USP54_TCYDSEDSGHLTDSECNQK.pdf]

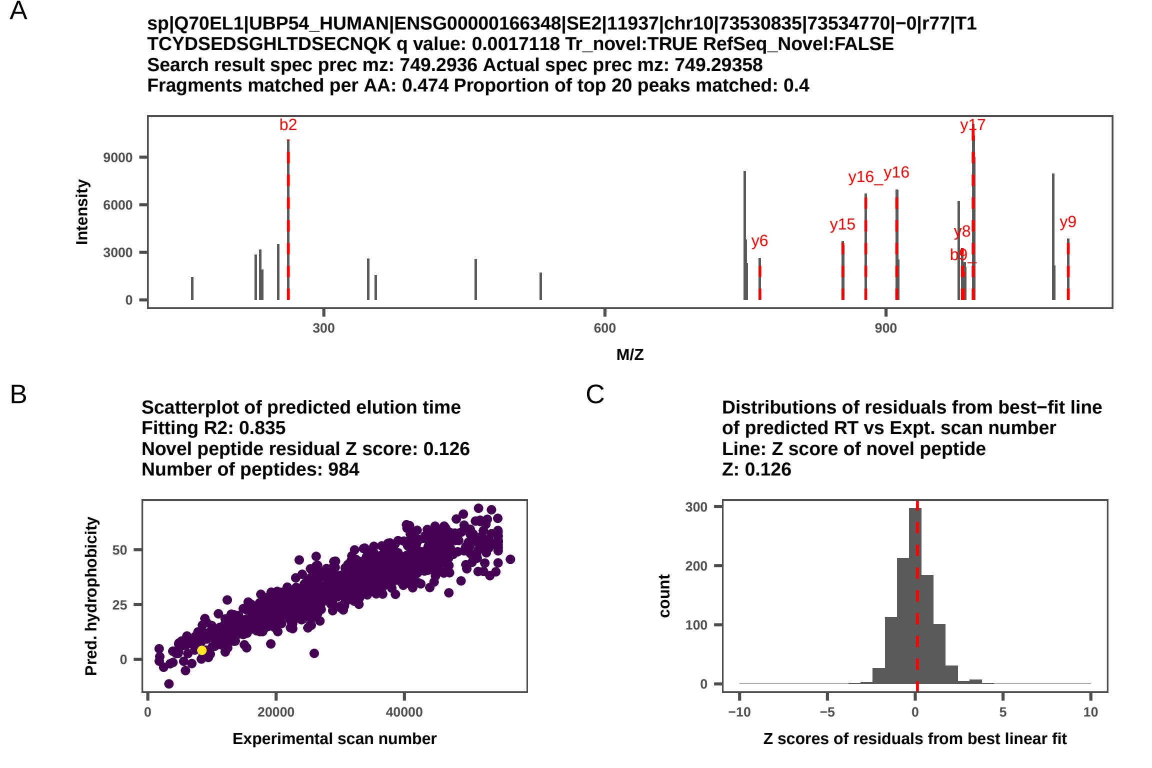
A
sp|Q70EL1|UBP54_HUMAN|ENSG00000166348|SE2|11937|chr10|73530835|73534770|−0|r77|T1
TCYDSEDSGHLTDSECNQK q value: 0.0017118 Tr_novel:TRUE RefSeq_Novel:FALSE
Search result spec prec mz: 749.2936 Actual spec prec mz: 749.29358
Fragments matched per AA: 0.474 Proportion of top 20 peaks matched: 0.4
Intensity
9000
6000
3000
0
b2
y6
y15
y16_y16
y8
b9_
y17
y9
300
600
900
M/Z
B
Scatterplot of predicted elution time
Fitting R2: 0.835
Novel peptide residual Z score: 0.126
Number of peptides: 984
Pred. hydrophobicity
50
25
0
0
20000
40000
Experimental scan number
C
Distributions of residuals from best−fit line
of predicted RT vs Expt. scan number
Line: Z score of novel peptide
Z: 0.126
count
300
200
100
0
−10
−5
0
5
10
Z scores of residuals from best linear fit
